# Tongue Tied after Shoulder Surgery: A Case Series and Literature Review

**DOI:** 10.1155/2019/5392847

**Published:** 2019-10-29

**Authors:** Molly B. Kraus, Rachel B. Cain, David M. Rosenfeld, Renee E. Caswell, Michael L. Hinni, Michael J. Molloy, Terrence L. Trentman

**Affiliations:** ^1^Mayo Clinic, Phoenix, AZ, USA; ^2^Southwestern Colorado Ear, Nose & Throat Associates, Durango, CO, USA

## Abstract

This article presents three cases of cranial nerve palsy following shoulder surgery with general anesthesia in the beach chair position. All patients underwent preoperative ultrasound-guided interscalene nerve block. Two cases of postoperative hypoglossal and one case of combined hypoglossal and recurrent laryngeal nerve palsies (Tapia's syndrome) were identified. Through this case series, we provide a literature review identifying postoperative cranial nerve palsies in addition to the discussion of possible etiologies. We suggest that intraoperative patient positioning and/or airway instrumentation is most likely causative. We conclude that the beach chair position is a risk factor for postoperative hypoglossal nerve palsy and Tapia's syndrome.

## 1. Introduction

With the advent of the beach chair position, cranial nerve palsies have been rarely reported following shoulder surgery [1–11]. Historically, shoulder surgery was performed in the lateral decubitus position with a traction apparatus. Postoperative nerve injuries to the brachial plexus were reported and largely attributed to the traction apparatus [12]. In the beach chair position, a traction apparatus is unnecessary, making postoperative brachial plexus nerve injury a rare occurrence.

We present a series of three cases of cranial nerve palsy following shoulder surgery that occurred within a three-month timeframe. All patients underwent ipsilateral preoperative ultrasound-guided interscalene brachial plexus block with local anesthetic. The surgeries included two arthroscopic rotator cuff repairs and a total shoulder arthroplasty, all in the beach chair position. Affected nerves included ipsilateral hypoglossal, recurrent laryngeal, and contralateral hypoglossal.

This paper will discuss the possible mechanisms of injury resulting in postoperative hypoglossal and recurrent laryngeal nerve palsy in this clinical setting. In addition, we provide a literature review and discussion of postoperative cranial nerve palsy.

### 1.1. Case 1

**Figure 1:**
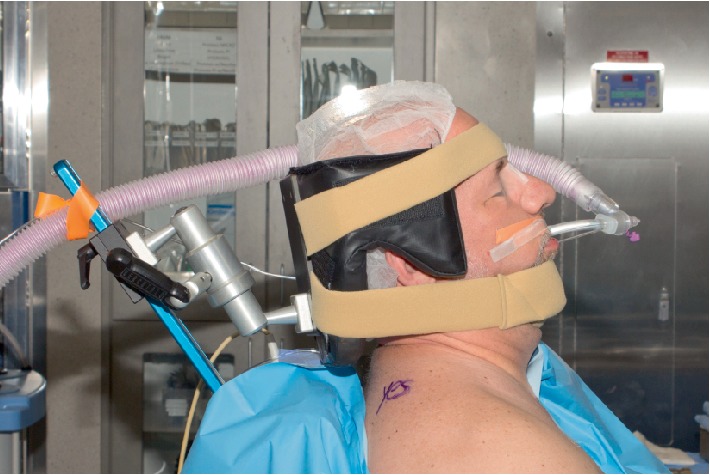
Positioning of head and neck during the beach chair position, secured via soft Velcro strap across forehead and around the chin.

**Figure 2:**
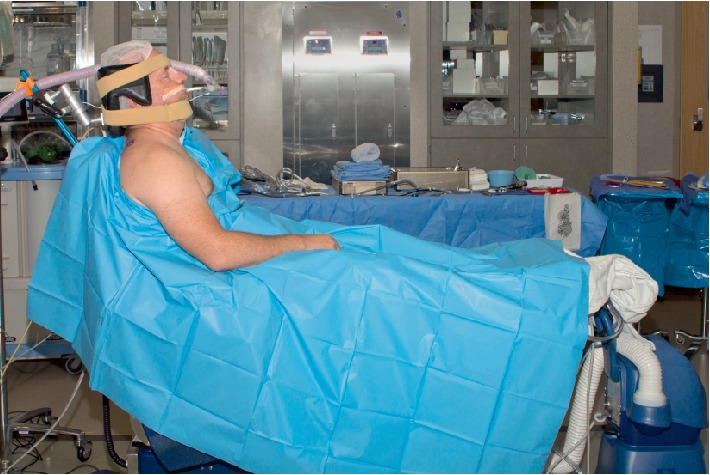
A typical beach chair position at our institution.

A 66-year-old 77-kilogram (kg) male with a history of gastroesophageal reflux and hypertension underwent arthroscopic repair of a right rotator cuff tear. Uncomplicated preoperative interscalene block was performed with real-time ultrasound guidance by an experienced anesthesiologist using a 22 gauge needle and thirty milliliters of 0.5% ropivacaine. Induction was with propofol, fentanyl, and succinylcholine. Mask ventilation was easy and uneventful. A MacIntosh blade was used and vocal cord visualization was a modified Cormack–Lahane grade 1, with subsequent successful placement of a 7.5 cm endotracheal tube (ETT) with one attempt [13]. No cricoid pressure was applied. The patient was positioned in the upright beach chair position. The surgical team supported the neck with a padded headrest in a neutral alignment and secured with a chinstrap and a second strap across the forehead. Chair angle was 60 degrees with hips and knees flexed. Surgical technique was standard with operative time of eighty minutes (Figures 1 and 2).

The following morning the patient was noticeably dysarthric. Examination revealed notable tongue weakness with rightward deviation on protrusion. The remaining neurologic and cranial nerve exam appeared normal. The preoperative block had resolved and he had normal upper extremity sensation. The neurology service was consulted and rendered a diagnosis of isolated right hypoglossal nerve palsy. Eight days after surgery, the patient noticed marked improvement in his symptoms, except slight tongue deviation to the right. All symptoms resolved by six weeks postoperatively (Table 1).

### 1.2. Case 2

A 69-year-old, 93-kg male with past medical history significant for coronary artery disease, obstructive sleep apnea, gastroesophageal reflux, and dyslipidemia underwent left arthroscopic rotator cuff repair. Preoperative interscalene block was performed as described above. Induction was with propofol, fentanyl, and rocuronium. Mask ventilation was easy and intubation was uncomplicated in a single attempt with an Phillips blade and an 8.0 cm ETT. The glottis was anterior and a Corrmack–Lahane grade 2b arytenoid view was obtained. The patient was positioned in beach chair as described in case 1. Routine surgery followed and concluded at eighty minutes.

The patient noted dysphonia and dysphagia immediately following surgery but was otherwise stable for discharge. On his surgical follow-up he continued to have dysphonia and dysphagia and was referred to otorhinolaryngology (ENT). On postoperative day 8, he was evaluated by ENT, he had a whispering voice and leftward deviation of his tongue. Flexible laryngoscopy showed left true vocal fold immobility, fixed at a 45 degree position with inability to approximate the cords with attempted adduction. Combined left hypoglossal and left recurrent laryngeal nerve palsy was diagnosed. The patient had difficulty swallowing and fear of oral intake due to sensation of aspiration, which resulted in unintentional weight loss of fifteen pounds. Speech therapy evaluation led to a formal swallow study, which was normal. Because both cranial nerves X and XII were affected, a CT angiogram was obtained to rule out dissection or aneurysm, and was negative. He subsequently underwent office-based medialization with hyaluronic acid to bring the immobile left vocal fold to a midline position.

ENT evaluation seven weeks postoperative with flexible laryngoscopy showed continued immobility of the left true vocal fold. The vocal fold was in the midline position allowing for complete glottic closure with phonation. This patient reported significant improvement in voice and swallowing with full range of motion of his tongue without deviation on protrusion. Ten weeks after surgery, the patient reported his tongue, swallowing, and voice had all returned to normal. At that point, flexible laryngoscopy showed slight improvement in left true vocal cord mobility, though still hypomobile, and excellent glottic closure (Table 1).

### 1.3. Case 3

A 75-year-old 87-kg male with past medical history of hypertension, gastroesophageal reflux, dyslipidemia, and chronic shoulder pain underwent a right total shoulder arthroplasty for degenerative arthritis. Preoperative interscalene block was performed as above. Induction was with propofol, fentanyl, lidocaine, and rocuronium. Mask ventilation and intubation was uncomplicated in one attempt with a Miller blade and a 7.5 cm ETT. Anesthesia was maintained with sevoflurane in oxygen/air. The patient was positioned in the beach chair position at 45 degrees. Routine surgery followed with sixty-seven minutes operative time.

In recovery, the patient noted dysarthria, difficulty swallowing, and a subjectively heavy tongue. He was evaluated by the anesthesia team and observed. When his symptoms were not completely resolved at his postoperative follow-up, his surgeon obtained ENT evaluation on postoperative day 11. Exam revealed leftward deviation of the tongue with persistent glossal weakness. Left hypoglossal nerve palsy was diagnosed. Three weeks postoperatively, the patient continued to have persistent left sided deviation but improved bilateral mobility. Six weeks later, his symptoms completely resolved (Table 1).

## 2. Discussion

The hypoglossal nerve, or twelfth cranial nerve, is purely a motor nerve responsible for innervating the extrinsic and intrinsic muscles of the tongue. Functions include the articulation of speech and the act of swallowing. Altered function results in slurred speech and difficulty with mastication. Patients with hypoglossal palsy describe the tongue as thick, heavy, or clumsy. To test the status of the nerve, a patient is asked to protrude his or her tongue. If there is a unilateral loss of function, the tongue will deviate to the ipsilateral side on protrusions, whereas at rest, the tongue will deviate to the contralateral side of the injury [16]. Injury to this nerve was noted in Cases 1 and 3.

The vagus nerve, or tenth cranial nerve, innervates the vocal folds via the recurrent laryngeal nerve branch. Our case series included hypoglossal and recurrent laryngeal nerve injuries following shoulder surgery. The combination of hypoglossal and recurrent laryngeal nerve injuries is called Tapia's syndrome. This results in an adducted vocal cord causing hoarseness, unilateral paralysis of the soft palate, and ipsilateral deviation of the tongue [15]. This was the diagnosis in Case 2.

Based on our observations and using information from the medical literature, several potential causes were identified: an injury related to the interscalene block, intubation trauma, compression by the ETT or laryngeal mask airway (LMA) cuff, positioning, or a combination of etiologies.

A literature search was conducted on PubMed databases from 1947 to September 2018. The keywords “Tapias syndrome”, “hypoglossal nerve palsy”, “recurrent laryngeal nerve palsy” were used. Only case reports on adults and written in English were included. Reference lists of cases identified were also used to find additional cases. In all, 28 cases of postoperative hypoglossal nerve palsy and 18 cases of postoperative Tapia's syndrome were identified. Twelve of these case reports were following shoulder surgery.

### 2.1. Interscalene Block

**Figure 3:**
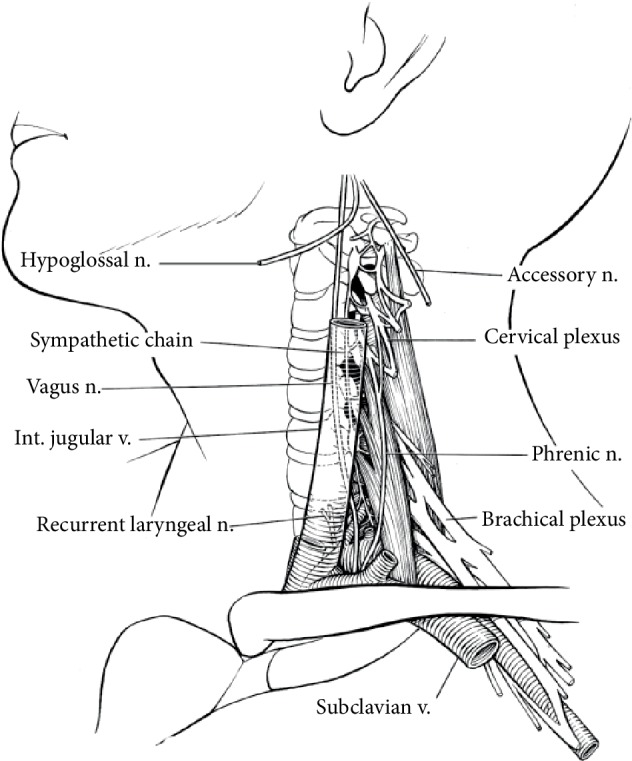
Hypoglossal, vagus, recurrent laryngeal nerves pictured. The brachial plexus between anterior and middle scalene is far caudal from the hypoglossal nerve.

Could these neurapraxias be related to an interscalene block where direct nerve injury is a known, although rare, complication? The brachial plexus at the interscalene block site in the inferolateral neck is positioned inferior and distant from the hypoglossal nerve (Figure 3). While the recurrent laryngeal nerve traverses closer to the brachial plexus, and since it was injured in conjunction with the hypoglossal nerve, the insult likely occurred where these two nerves are in close proximity, which is distant from the brachial plexus. The brachial plexus passes between the anterior and middle scalene muscles before descending under the clavicle. An interscalene block is performed using an ultrasound to identify the subclavian artery and brachial plexus. The brachial plexus is then traced cranially until the nerve trunks are seen as a “snowman” pattern between the anterior and middle scalene. Local anesthetic is then deposited around the identified nerve trunks.

In all three of our cases preoperative interscalene blocks were performed using ultrasound. Three different anesthesiologists performed the preoperative blocks and conducted the anesthesia in a team care model (Table 1). The interscalene block is unlikely causative, considering the injuries were not all ipsilateral. Another factor is the anatomic injury site, considering the hypoglossal nerve is typically cephalad and anterior. This conclusion is consistent with case reports in the literature.

We have identified seven manuscripts describing eight cases [1–5, 11] of isolated hypoglossal nerve palsy after shoulder surgery. Regional anesthesia was not performed in any of these cases and thus not causative. Four additional articles were identified with Tapia's syndrome following shoulder surgery [6–9]. Two of those case reports included preoperative interscalene blocks as part of the anesthetic management [8, 9].

Johnson et al. described a case of Tapia's syndrome following left shoulder Mumford procedure (distal clavicle excision) in a patient who received an interscalene block [9]. They discount the interscalene block as the mechanism of injury because the injection site for the block is caudal to the anatomic crossing of the hypoglossal and vagus nerves at the carotid sheath. They postulate that their patient sustained a dissection of the ascending pharyngeal branch of the carotid artery, which provides the exclusive blood supply to cranial nerves X and XII. They suspected that a dissection could happen after minor trauma, including block placement. However, vascular imaging performed three days later failed to identify a dissection. In our case of Tapia's syndrome (Case 2), a CT angiography was performed on postoperative day 8 and failed to identify a dissection or any other cause of cranial nerve injury.

In Wadelek et al.'s case of Tapia's syndrome following shoulder arthroscopy with interscalene block, an MRI was performed which revealed a submucosal hematoma at the tongue base [8]. The palsy was on the opposite side of the hematoma. They postulated that the hematoma may be related to an oversized LMA causing excessive pressure on the vagus and hypoglossal nerves.

Unrelated isolated hypoglossal and recurrent laryngeal nerve injury in the same surgery from a block is highly unlikely.

### 2.2. Airway Manipulation

Upon review of the literature, several cases concluded that airway manipulation and instrumentation may lead to injury of the hypoglossal and recurrent laryngeal nerves (see Tables 2 and 3). Mask ventilation, oral airways, LMA cuffs, ETT cuffs, laryngoscopy or cricoid pressure were all postulated as potential causes of injury. In our series all three patients were described as “easy” mask ventilation and the trachea was intubated with one attempt. The intubations of Cases 1 and 3 were described as a full view. In Case 2, the patient had an anterior glottis; however, the provider was able to visualize the arytenoids. None required multiple attempt laryngoscopy or cricoid pressure. The cuff pressure was not confirmed with a manometer in any of our three cases.

During intubation, the patient's tongue is pushed forward and the neck can be extended, which results in traction on the hypoglossal nerve [10]. Michel and Brusis in a cadaver study reported a 1.3 cm stretch on the hypoglossal nerve during laryngoscopy [17]. Even more stretch was found with extension of the neck, and more aggressive laryngoscopy [17]. Streppel et al. reported a case of hypoglossal nerve injury following rhinoplasty with general endotracheal anesthesia. They concluded that a short compression of the nerve between the Macintosh laryngoscope blade and a calcified ligamentum styloideum resulted in injury [18]. Direct compression of the nerve beneath the angle of the mandible with finger pressure during mask ventilation may also cause injury [4].

In Tapia's syndrome, mechanical stress on both the hypoglossal and recurrent laryngeal nerves is likely the etiology. The two nerves are in close proximity at the base of the tongue and in the pyriform fossa. They also cross the lateral prominence of the anterior surface of the transverse process of C1. The nerves may be compressed between the ETT and a stiff structure such as greater cornu of the hyoid bone, thyroid cartilage, or cervical vertebrae [19]. Compression may also be caused by a combination of excessive throat pack and the ETT cuff [20]. Throat packs were implicated in several cases of Tapia's syndrome with throat packs used in 8 of 18 cases (44%) (Table 4). Rubio-Nazabal et al. concluded that unnoticed, prolonged overinflation of the endotracheal cuff resting just below the cords was to blame for a case of bilateral nerve compression [21].

Two case reports of Tapia's syndrome and isolated hypoglossal nerve palsy suggested that the ETT was left attached to a pressure gauge to maintain cuff pressure <20 cm H_2_O [4, 22]. The use of nitrous oxide was not clearly reported in all cases (Table 4). Nitrous oxide is well known to enter and expand air spaces and has been reported to increase LMA cuff pressures by as much as 38% in thirty minutes [23]. Its use may contribute to cranial nerve injury; however, it was not used in many reported cases nor our current series.

### 2.3. Positioning

In our review, patient positioning was not clearly described in all cases. When it was reported, the beach chair, supine, and semi-supine positions were most common. While many reports provided varying possible mechanisms of injury, the beach chair position was used in 9 of 12 of cranial nerve palsy cases following shoulder surgery (Table 2). Overall, this position was used in 9 of 46 cases (19.5%) of postoperative cranial nerve palsy. We identified 28 total cases of postoperative hypoglossal nerve palsy overall, with shoulder surgery accounting for 8 of these 28 (28%) of hypoglossal nerve palsy, and 12 of 46 (26%) of the cases of Tapia's syndrome. While intubation and airway manipulation remain a potential cause of postoperative cranial nerve palsy, positioning may also play a role in these injuries following shoulder surgery.

The hypoglossal nerve exits the skull and proceeds caudally between the internal carotid artery and internal jugular vein, and passes inferiorly to the angle of the mandible. While positioned in the beach chair position, a head strap is used to secure the head in a neutral position. Placing the strap too tightly at the angle of the mandible or manipulation during surgery, can cause direct compression or hyperextension to the hypoglossal nerve [50]. It is plausible that while wanting to secure the head in a secure neutral position, overzealous tightening of the head strap and direct compression of the hypoglossal nerve could cause injury. Menorca et al. [51] discuss possible etiologies of peripheral nerve damage secondary to direct compression. Theories include narrowing of openings leads to increased pressure at that site, compressing blood vessels and leading to nerve ischemia or decreased venous return secondary to local pressure which can lead to venous stasis. In this state, extraneural edema may form over time with subsequent fibrous and scar tissue around the nerve and eventual intraneural edema.

Surgical manipulation, such as a surgical assistant's elbow, can compress the mandible angle during surgery [52]. In the beach chair position even a small change in position may modify the angle of the trunk in relation to the headrest. This can cause hyperextension of the neck or unintended compression, leading to hypoglossal nerve injury. Too much flexion or extension on the headrest could also lead to stretch or compression (Figure 1). The hypoglossal nerve becomes superficial at the angle of the mandible and is susceptible to injury. This may occur from initial positioning or a change in position of the neck during the case [1].

Boisseau, et al. concluded that positioning was the cause of a patient's postoperative Tapia's syndrome as when the surgical drapes were removed at the conclusion of the case the patient's head was noted with a pronounced right lateral flexion [7]. When the patient is positioned with leftward head rotation for surgery on the right shoulder, positional injury can occur due to nerve compression and traction [6]. Generally, poor positioning during surgery can cause vascular, neurological or tissue damage. No report has been published providing an anatomical explanation for Tapia's syndrome in relation to shoulder surgery. If the head is flexed laterally, the endotracheal tube may exert pressure in a localized area at the crossing of the vagal and hypoglossal nerves. When a patient is maintained in an upright position for surgery, the patient is particularly susceptible to head misplacement. Maintaining head alignment in field avoidance surgeries can be difficult as the body is draped and not easily accessible [7].

### 2.4. Recovery

Fortunately, postoperative cranial nerve palsies generally have a good prognosis. They are often mild injuries to the myelin sheath that result from ischemia or mechanical compression. Neurapraxias typically resolve within three months, as is the situation with our three patients. Axonotmesis is more severe and caused by a crush injury or extreme traction on the nerve and has a more tenuous recovery. Complete recovery of the hypoglossal nerve function, if occurs, is expected within the first six months. Interventions are largely unnecessary. Several case reports describe the use of corticosteroids for several days after injury; however this is not supported in the literature. Patients may benefit from speech therapy to improve their ability to swallow by training to chew and manipulate food on the noninvolved side [24].

## 3. Conclusion

Cranial nerve palsy following shoulder surgery is rare. In our series, both ipsilateral and contralateral cranial neurapraxia occurred, which is consistent with previous reports in the literature. Intraoperative patient positioning and airway instrumentation, as opposed to preoperative regional nerve block or operative technique, is the most likely causative factor.

Based on our literature review, we conclude that the beach chair position is a risk factor for postoperative hypoglossal nerve palsy or Tapia's syndrome. To prevent cranial nerve injury, the anesthesiologist and surgical team must be vigilant in positioning. In the beach chair position in particular we must respect the craniospinal axis. Head position should be verified for neutrality on initial positioning and frequently during the procedure. The anesthesiologist should monitor and minimize endotracheal cuff pressures, and avoid prolonged laryngoscopy.

## Figures and Tables

**Table 1:** Summary of our cases.

Case	Surgery	Position		Airway	Injury	IS block	Nitrous oxide used
1	Shoulder arthroscopy	Beach chair	Oral ETT		Ipsilateral hypoglossal	Yes	No
2	Shoulder arthroscopy	Beach chair	Oral ETT		Ipsilateral Tapia's syndrome	Yes	No
3	Total shoulder	Beach chair	Oral ETT		Contralateral hypoglossal	Yes	No

**Table 2:** Case reports of cranial nerve palsy after shoulder surgery.

First author	Surgery	Position	Airway	Injury	IS block	Nitrous oxide used	Determined cause
Mullins [1]	Open repair or rotator cuff	Beach chair	Oral ETT	Contralateral hypoglossal	No	NA	Positioning
Hwang [2]	Open repair of humeral fracture	Beach chair	Oral ETT	Hypoglossal	No	NA	Positioning
Rhee [3]	Arthroscopy	Beach chair	Oral ETT	Contralateral hypoglossal	No	NA	Positioning
Rhee [3]	Arthroscopy	Beach chair	Oral ETT	Contralateral hypoglossal	No	NA	Positioning
Hung [4]	Arthroscopy	Semi-beach-chair	Oral ETT	Ipsilateral hypoglossal	No	No	Intubation or mask ventilation
Nagai [5]	Total shoulder	Right lateral	LMA	Contralateral hypoglossal	No	Yes	LMA, N2O, change in position
Cogan [6]	Arthroscopy	Beach chair	Oral ETT	Tapia's syndrome	No	NA	Positioning
Boisseau [7]	Arthroscopy	Beach chair	Oral ETT	Ipsilateral Tapia's syndrome	No	No	Positioning
Wadelek [8]	Arthroscopy	Semi-supine	LMA	Tapia's syndrome	Yes	No	LMA and positioning
Johnson [9]	Shoulder Mumford Procedure	NA	NA	Ipsilateral Tapia's syndrome	Yes	NA	Dissection of the ascending pharyngeal artery
Dziewas [10]	Arthroscopy	NA	Oral ETT	Hypoglossal	No	NA	laryngoscopy
Haslam [11]	Total shoulder	Beach chair	Oral ETT	Contralateral hypoglossal	Yes	NA	ETT

NA = not available; IS = interscalene block.

**Table 3:** Additional case reports of postoperative Tapia's syndrome.

First Author	Surgery	Position	Airway	Throat Pack	Nitrous Oxide Used	Determined Cause
Ota [19]	Le Fort osteotomy and genioplasty	NA	transnasal ETT	Yes	NA	Intubation and position
^∗^Cinar [22]	Rhinoplasty	Semi-recumbent	Oral ETT	Yes	No	Excessive cuff pressure
Poveda [20]	Rhinoplasty	Semi-supine	Oral ETT	Yes	No	Throat pack and positioning
Yavuzer [39]	Septorhinoplasty	Unspecified	Oral ETT	Yes	NA	ETT
^∗^Una [40]	Anterior mediastinotomy	NA	Oral ETT fiberoptic intubation	No	NA	ETT
Nalladaru [41]	CABG	Supine	Oral ETT	No	NA	Intubation or positioning
Park [42]	Posterior cervical spine	Concord position	Oral ETT	NA	NA	Positioning
Lykoudis [43]	Rhinoplasty	NA	Oral ETT	Yes	NA	Throat pack
Kashyap [44]	Repair of mandibular fracture	NA	Nasal ETT	NA	NA	Unclear
Lim [45]	Cervical laminoplasty	Prone	Oral ETT	No	No	ETT and positioning
Sotiriou [46]	CABG	Supine	Oral ETT	No	NA	positioning
Bakhshaee [47]	Rhinoplasty	NA	Oral ETT	Yes	Yes	Intubation, ETT, throat pack, controlled hypotension
Varedi [48]	Zygomatic arch repair	NA	Transnasal ETT	Yes	NA	Unclear
Ghorbani [49]	Septorhinoplasty	Supine with head elevated	Oral ETT	Yes	NA	ETT, throat pack, position

^∗^= bilateral; NA = not available; IS = interscalene block.

**Table 4:** Additional case reports of postoperative hypoglossal nerve palsy.

First author	Surgery	Position	Airway	Throat pack	Nitrous oxide used	Determined cause
^∗^Rubio-Nazabal [21]	Open AAA repair	NA	Oral ETT	No	NA	Excessive cuff pressure
Streppel [18]	Sinus surgery	NA	Oral ETT	No	NA	Calcified ligamentum stylohoideum and intubation
Dearing [26]	Molar surgery	NA	Nasal ETT	Yes	NA	Intubation or throat pack
Dwiewas [10]	Esophageal resection	NA	Oral ETT	No	NA	laryngoscopy
Evers [16]	Trans-spenoidal hypophysectomy	Supine	Oral ETT	Yes	Yes	Intubation or throat pack
Ulusoy [27]	Septohinoplasty	Semi-supine	Oral ETT	Yes	Yes	Intubation or mask ventilation
Venkatesh [28]	Craniotomy	NA	Oral ETT	No	Yes	Accidental extubation with inflated cuff
King [29]	Removal of rush pins	NA	LMA	No	Yes	LMA
Lopes [30]	Breast reconstruction	Lateral decubitus and sitting	Oral ETT	No	No	Positioning
Lopes [30]	Breast reduction	Semi-sitting and dorsal decubitus	Oral ETT	No	No	Positioning
Yelken [25]	Open septoplasty	NA	Oral ETT	Likely but unspecified	NA	Intubation and arnold chiari
Michel [17]	Tonsillectomy	NA	Oral ETT	NA	NA	laryngoscopy
Lo [31]	ORIF humerus	NA	LMA	No	NA	Malpositioned LMA
^∗^Stewart [32]	Knee arthroscopy	NA	LMA	No	Yes	Excessive cuff pressure
Baumgarten [33]	Septoplasty	NA	Oral ETT	NA	NA	Intubation
Trumpelmann [34]	ORIF tibial plateau fracture	NA	LMA	NA	Yes	LMA and nitrous oxide
Takahoko [35]	Hallux valgus correction	Supine	LMA	No	Yes	LMA and nitrous oxide
Al-Benna [36]	Breast augmentation	30 degree elevated supine	Oral ETT	No	Yes	ETT
Slaats [37]	Exploratory laparoscopy	Supine	Oral ETT	No	NA	Intubation, hematoma near nerve
^∗^Sommers [38]	Removal of scar tissue behind ears	Extreme side rotation of head	LMA	No	No	Positioning

^∗^= bilateral; NA = not available; IS = interscalene block.

## References

[1] Mullins R. C., Drez Jr D., Cooper J. (1992). Hypoglossal nerve palsy after arthroscopy of the shoulder and open operation with the patient in the beach-chair position. A case report. *The Journal of Bone and Joint Surgery*.

[2] Hwang J.-Y., Won H.-R., Hong Y.-H., Mun S.-K. (2010). Isolated hypoglossal nerve palsy following open surgery in the beach-chair position under general anesthesia: a case report. *International Journal of Pediatric Otorhinolaryngology Extra*.

[3] Rhee Y. G., Cho N. S. (2008). Isolated unilateral hypoglossal nerve palsy after shoulder surgery in beach chair position. *Journal of Shoulder and Elbow Surgery*.

[4] Hung N. K., Lee C. H., Chan S. M. (2009). Transient unilateral hypoglossal nerve palsy after orotracheal intubation for general anesthesia. *Acta Anaesthesiologica Taiwanica*.

[5] Nagai K., Sakuramoto C., Goto F. (1994). Unilateral hypoglossal nerve paralysis following the use of the laryngeal mask airway. *Anaesthesia*.

[6] Cogan A., Boyer P., Soubeyrand M., Hamida F. B., Vannier J.-L., Massin P. (2011). Cranial nerves neuropraxia after shoulder arthroscopy in beach chair position. *Orthopaedics & Traumatology: Surgery & Research*.

[7] Boisseau N., Rabarijaona H., Grimaud D., Raucoules-Aime M. (2002). Tapia’s syndrome following shoulder surgery. *British Journal of Anaesthesia*.

[8] Wadelek J., Kolbusz J., Orlicz P., Staniaszek A. (2012). Tapia’s syndrome after arthroscopic shoulder stabilization under general anaesthesia and LMA. *Anaesthesiology Intensive Therapy*.

[9] Johnson T. M., Moore H. J. (1999). Cranial nerve X and XII paralysis after an interscalene brachial plexus block for a left shoulder mumford procedure. *Anesthesiology*.

[10] Dziewas R., Ludemann P. (2002). Hypoglossal nerve palsy as complication of oral intubation, bronchoscopy, and use of the laryngeal mask airway. *European Neurology*.

[11] Haslam B., Collins S. (2013). Unilateral hypoglossal neurapraxia following endotracheal intubation for total shoulder arthroplasty. *AANA Journal*.

[12] Rodeo S. A., Forster R. A., Weiland A. J. (1993). Neurologial complications due to arthroscopy. *Journal of Bone and Joint Surgery*.

[13] Yentils S. M., Lee D. J. H. (1998). Evaluation of an improved scoring system for the grading of direct laryngoscopy. *Anaesthesia*.

[14] Leblanc A. (2004). *Hypoglossal Nerve (XII) Encephalo-Peripheral Nervous System*.

[15] Schoenberg B. S., Massey E. W. (1979). Tapia’s syndrome the erratic evolution of an eponym. *Archives of Neurology*.

[16] Evers K. A., Eindhoven G. B., Wierda J. M. (1999). Transient nerve damage following intubation for trans-sphenoidal hypophysectomy. *Canadian Journal of Anesthesia*.

[17] Michel O., Brusis T. (1990). Hypoglossal nerve paralysis following tonsillectomy. *Laryngo-Rhino-Otologie*.

[18] Streppel M., Bachmann G., Stennert E. (1997). Hypoglossal Nerve Palsy as a Complication of Transoral Intubation for General Anesthesia. *Anesthesiology*.

[19] Ota N., Izumi K., Okamoto Y. (2013). Tapia's syndrome following the orthognathic surgery under general anaesthesia. *Journal of Oral and Maxillofacial Surgery, Medicine, and Pathology*.

[20] Tesei F., Poveda L. M., Strali W., Tosi L., Magnani G., Farneti G. (2006). Unilateral laryngeal and hypoglossal paralysis following rhinoplasty in general anesthesia:case report and review of the literature. *Acta Otorhinolaryngologica Italica*.

[21] Rubio‐Nazábal E., Marey‐Lopez J., Lopez‐Facal S., Alverez‐Perez P., Martinez‐Figueroa A., Rey del Corral P. (2002). Isolated bilateral paralysis of the hypoglossal nerve after transoral intubation for General Anesthesia. *Anesthesiology*.

[22] Cinar S. O., Seven H., Cinar U., Turgut S. (2005). Isolated bilateral paralysis of the hypoglossal and recurrent laryngeal nerves after transoral intubation for general anesthesia. *Acta Anaesthesiologica Scandinavica*.

[23] Lumb A. B., Wrigley M. W. (1992). The effect of nitrous oxide on laryngeal mask cuff pressure. In vitro and in vivo studies. *Anaesthesia*.

[24] Fritz M. A., Kang B. J., Timothy P. F., Bhatia N., Mandel S. (2014). Iatrogenic hypoglossal nerve palsy. *Practical Neurology*.

[25] Yelken K., Guven M., Kablan Y., Sarikaya B. (2008). Isolated unilateral hypoglossal nerve paralysis following open septoplasty. *British Journal of Oral and Maxillofacial Surgery*.

[26] Dearing J. (1998). Transient contralateral hypoglossal nerve palsy following third molar surgery under day-case general anaesthesia: a case report and review of the literature. *British Journal of Oral and Maxillofacial Surgery*.

[27] Ulusoy H., Besir A., Cekic B., Kosucu M., Geze S. (2013). Transient unilateral combined paresis of the hypoglossal nerve and lingual nerve following intubation anesthesia. *Brazilian Journal of Anesthesiology*.

[28] Venkatesh B., Walker D. (1997). Hypoglossal neuropraxia following endotracheal intubation. *Anaesthes Intensive Care*.

[29] King C., Street M. K. (1994). Twelfth cranial nerve paralysis following use of a laryngeal mask airway. *Anaesthesia*.

[30] Lopes G., Denoel C., Duester G., Docquier M. A. (2009). Two cases of isolated unilateral paralysis of hypoglossal nerve after uncomplicated orotracheal intubation. *Acta Anaesthesiologica Belgica*.

[31] Lo T. S. (2006). Unilateral hypoglossal nerve palsy following the use of the laryngeal mask airway. *Canadian Journal of Neurological Sciences*.

[32] Stewart A., Lindsay W. A. (2002). Bilateral hypoglossal nerve injury following the use of the laryngeal mask airway. *Anaesthesia*.

[33] Baumgarten V., Jalinski W., Bohm S., Galle E. (1997). Hypoglossal paralysis after septum correction with intubation anesthesia. *Anaesthesist*.

[34] Trümpelmann P., Cook T. (2005). Unilateral hypoglossal nerve injury following the use of a ProSeal™ laryngeal mask. *Anaesthesia*.

[35] Takahoko K., Iwasaki H., Sasakawa T., Suzuki A., Matsumoto H., Iwasaki H. (2014). Unilateral Hypoglossal Nerve Palsy after Use of the Laryngeal Mask Airway Supreme. *Case Reports in Anesthesiology*.

[36] Al-Benna S. (2013). Right hypoglossal nerve paralysis after tracheal intubation for aesthetic breast surgery. *Saudi Journal of Anaesthesia*.

[37] Slaats M., Potvin J., Vanlinthout L., Declau F. (2014). Unilateral hypoglossal nerve paralysis after general anthessia. *International Journal of Surgery*.

[38] Sommer M., Schuldt M., Runge U., Gielen-Wijffels S., Marcus M. A. (2004). Bilateral hypoglossal nerve injury following the use of the laryngeal mask without the use of nitrous oxide. *Acta Anaesthesiologica Scandinavica*.

[39] Yavuzer R., Başterzi Y., Æzköse Z., Yücel Demir H., Yilmaz M., Ceylan A. (2004). Tapia’s syndrome following septorhinoplasty. *Aesthetic Plastic Surgery*.

[40] Uña E., Gandia F., Duque J. (2009). Tongue paralysis after orotracheal intubation in a patient with primary mediastinal tumor: a case report. *Cases Journal*.

[41] Nalladar Z., Wessels A., Dupreez L. (2012). Tapia's syndrome – a rare complication following cardiac surgery. *Interactive CardioVascular and Thoracic Surgery*.

[42] Park C. K., Lee D. C., Park C. J., Hwang J. H. (2013). Tapia's Syndrome after Posterior Cervical Spine Surgery under General Anesthesia. *Journal of Korean Neurosurgical Society*.

[43] Lykoudis E. G., Seretis K. (2012). Tapia’s syndrome: an unexpected but real complication of rhinoplasty: case report and literature review. *Aesthetic Plastic Surgery*.

[44] Kashyap S. A., Patterson A. R., Loukota R. A., Kelly G. (2010). Tapia’s syndrome after repair of a fractured mandible. *British Journal of Oral and Maxillofacial Surgery*.

[45] Lim K. J., Kim M. H., Kang M. H. (2013). Tapia's syndrome following cervical laminoplasty -A case report-. *Korean Journal of Anesthesiology *.

[46] Sotiriou K., Balanika M., Anagnostopoulou S., Gomatos C., Karakitsos D., Saranteas T. (2007). Postoperative airway obstruction due to Tapiaʼs syndrome after coronary bypass grafting surgery. *European Journal of Anaesthesiology*.

[47] Bakshaee M., Bameshki A. R., Foroughipour M., Zaringhalam M. A. (2014). Unilateral recurrent laryngeal and hypoglossal nerve paralysis following rhinoplasty: a case report and review of the literature. *Iranian Journal of Toxicology*.

[48] Varedi P., Shirani G., Karimi A., Varedi P., Khiabani K., Bohluli B. (2013). Tapia syndrome after repairing a fractured zygomatic complex: a case report and review of the literature. *Journal of Oral and Maxillofacial Surgery*.

[49] Ghorbani J., Dabir S., Givehchi G., Najafi M. (2014). Co-presentation of Tapia’s syndrome and pressure alopecia—a rare event after septorhinoplasty: a case report and literature review. *Acta Anaesthesiologica Taiwanica*.

[50] Kim C. J., Oh H. S., Park J.-J., Chung M. Y. (2016). Cranial nerve XII (hypoglossal nerve) palsy after arthroscopic shoulder surgery under general anesthesia combined with sono-guided interscalene brachial plexus block A case report. *Anesthesia and Pain Medicine*.

[51] Menorca R. M., Fussell T. S., Elfar J. C. (2013). Nerve physiology: mechanisms of injury and recovery. *Hand Clinics*.

[52] Hung N. K., Lee C. H., Chan S. M. (2009). Transient unilateral hypoglossal nerve palsy after orotracheal intubation for general anesthesia. *Acta Anaesthesiologica Taiwanica*.

